# Centrilobular emphysema combined with pulmonary fibrosis results in improved survival: a response

**DOI:** 10.1186/1755-1536-4-16

**Published:** 2011-07-25

**Authors:** Vincent Cottin, Jean-François Cordier, Athol U Wells

**Affiliations:** 1 Hospices Civils de Lyon, Hôpital Louis Pradel, Service de Pneumologie - Centre de Référence National des Maladies Pulmonaires Rares, Lyon, France; 2 Université de Lyon, Université Lyon I, INRA, UMR754 INRA-Vetagrosup EPHE IFR 128, Lyon, France; 3 Royal Brompton Hospital, London, UK

## Abstract

Better survival in combined pulmonary fibrosis and emphysema than in lone pulmonary fibrosis: bias or reality? A response to Centrilobular emphysema combined with pulmonary fibrosis results in improved survival by Todd *et al*., Fibrogenesis & Tissue Repair 2011, 4:6.

Please see related letter http://fibrogenesis.com/content/4/1/17

## 

We read with great interest the somewhat thought-provoking article by Todd *et al*. [1], which addressed the prognosis of patients with combined pulmonary fibrosis and emphysema (CPFE). The authors found that survival in CPFE was better than that of patients with idiopathic pulmonary fibrosis (IPF) without emphysema or with minimally extensive emphysema ('lone fibrosis'), in striking contrast to previous results [2-4]. From these counterintuitive results, it is argued that tobacco-smoking-induced pulmonary inflammation may be protective against the progression of fibrosis. However, in reaching this conclusion, we feel the authors have failed to sufficiently consider a number of potential biases that are evident from the stated data.

Firstly, amongst biopsied patients, fibrotic non-specific interstitial pneumonia (NSIP) was more prevalent in association with CPFE (6 of 20, 30%) than in the remaining groups (7 of 53, 13%). If these proportions are extrapolated to the whole cohort, including non-biopsied cases, the prevalence of fibrotic NSIP was significantly higher in the CPFE cases. Furthermore, this imbalance may have been amplified by the inclusion of non-biopsied cases: it is well recognized that NSIP when combined with emphysema may mimic honeycombing and can, therefore, be confused with genuine IPF [5]. A bias towards NSIP would also explain the unexpectedly higher female rate in the CPFE group.

Secondly, the way in which transplantation was handled in the survival analysis is very open to question. It has been usual to censor at date of transplantation, and not to handle a transplant event as a death. The authors point out that the rate in transplantation did not differ between groups. However, as at least a third of 'deaths' (n = 34) were transplant episodes, it is likely that this way of handling transplant events as deaths distorted the Kaplan-Meier curves.

Thirdly, potential lead-time bias was a significant concern in this study. For example, patients with CPFE were more frequently ever-smokers than their counterparts with lone fibrosis, and conceivably were more likely to suffer from chronic bronchitis and thus to seek medical advice or to draw medical attention. A better outcome in patients with CPFE compared with patients with lone fibrosis may therefore reflect earlier identification of disease.

Lastly and most importantly, patients with CPFE (pulmonary fibrosis with significant emphysema) and those with lone fibrosis had comparable carbon monoxide diffusion capacity (DLco) in the study by Todd *et al*. [1]. As DLco reflects the combined impact of emphysema and fibrosis [6], it follows that patients with more extensive emphysema must have had less extensive fibrosis, to end up with the same average level of DLco. In other words, based on DLco levels, patients with CPFE must have had significantly less extensive fibrosis than the lone fibrosis group.

In essence, Todd *et al*. report better survival, using a highly controversial outcome analysis, in a patient sub-group (CPFE) with a higher prevalence of fibrotic NSIP and much less extensive fibrosis. These confounding factors would seem to adequately account for the observed group differences. Thus, whilst Todd *et al*. should be commended for addressing the question of the relative prognosis of CPFE versus IPF, the biases discussed here make it difficult to reach clear conclusions. Such studies stimulate research by suggesting unreliable yet intriguing conclusions. Adequately conducted studies with adjustment for severity, and extent of fibrosis, and control of confounding factors are now needed to address the unresolved issue of whether CPFE and lone IPF may differ in outcome.

## Competing interests

The authors declare that they have no competing interests.

## Authors' contributions

VC conceived the study and drafted the manuscript; AW designed the study and contributed to draft the manuscript; JFC designed the study and contributed to draft the manuscript; all authors read and approved the final manuscript.
